# Green synthesis of silver nanoparticles using the extract of spent coffee used for paper-based hydrogen peroxide sensing device

**DOI:** 10.1038/s41598-022-22067-6

**Published:** 2022-11-22

**Authors:** Natwat Srikhao, Artjima Ounkaew, Pornnapa Kasemsiri, Somnuk Theerakulpisut, Manunya Okhawilai, Salim Hiziroglu

**Affiliations:** 1 Department of Chemical Engineering, Faculty of Engineering, Khon Kaen University, Khon Kaen, 40002 Thailand; 2 Energy Management and Conservation Office, Faculty of Engineering, Khon Kaen University, Khon Kaen, 40002 Thailand; 3 Center of Excellence in Responsive Wearable Materials, Chulalongkorn University, Bangkok, 10330 Thailand; 4 Research Unit in Polymeric Materials for Medical Practice Devices, Department of Chemical Engineering, Faculty of Engineering, Chulalongkorn University, Bangkok, 10330 Thailand; 5 Metallurgy and Materials Science Research Institute, Chulalongkorn University, Bangkok, 10330 Thailand; 6 Department of Natural Resource Ecology and Management, Oklahoma State University, Stillwater, OK 74078 USA

**Keywords:** Materials science, Nanoscience and technology

## Abstract

Hydrogen peroxide (H_2_O_2_) has attracted considerable attention for use as a disinfectant ingredient for various applications over the decades. The use of H_2_O_2_ within the safety regulations can avoid its toxicity to human health and the environment. In this study, a paper-based sensor containing green-synthesized silver nanoparticles (P-AgNPs) was developed for use in a smartphone in the determination of the H_2_O_2_ concentration. In the synthesis process, an extract of spent coffee grounds was used as a bioreducing agent. The effects of reaction time and silver nitrate (AgNO_3_) concentration on the green synthesis of silver nanoparticles (AgNPs) were investigated. The optimum conditions for the preparation of P-AgNPs were determined to be 100 mM AgNO_3_ (P-AgNPs-100) and 15 h synthesis time. The P-AgNPs-100 sensor exhibited high sensitivity with a detection limit of 1.26 mM H_2_O_2_, which might be suitable for the detection of H_2_O_2_-based household and beverage sanitizers. The H_2_O_2_ detection capability of P-AgNPs-100 was comparable to that of a commercial strip sensor. Furthermore, P-AgNPs-100 had a detection efficiency of more than 95% after long-term storage for 100 days.

## Introduction

Hydrogen peroxide (H_2_O_2_) has become more crucial to humanity since the breakout of COVID-19. H_2_O_2_ can be used as a disinfectant ingredient in nasal spray^1^, beverages^2^, and household cleansers^3^. Apart from these applications, H_2_O_2_ has also been widely used in textile, water treatment, and food processes^4^. However, the misuse of H_2_O_2_ preparations may be toxic to human health and the environment. At low concentrations (i.e., approximately 3% solution), H_2_O_2_ can cause mild gastrointestinal irritation, mild mucosal irritation, and vomiting^5^. Therefore, the use of H_2_O_2_ for various applications needs to be monitored. Among several analytical methods for H_2_O_2_ detection, optical sensors based on the surface of noble metal nanoparticles (NPs) known as localized surface plasmon resonance (SPR) have gained attention from researchers because NPs, such as gold, platinum, and silver, exhibit high absorption coefficients and high surface-to-volume ratios^6–9^. Silver nanoparticles (AgNPs) are one of the NPs that have attracted considerable interest for optical sensor applications because of their superior plasmonic characteristic^8^. Recently, a functionalized paper containing AgNPs was developed as a paper-based sensor that could be easily handled^10^. The prepared paper-based sensor containing carbon dot functionalized paper coupled with AgNPs could stably detect H_2_O_2_ for 4 weeks with a detection limit of 1.38 µM^11^. For AgNP production, the green synthesis of AgNPs is an environmentally friendly process that has attracted considerable attention^12^. The properties of green-synthesized AgNPs, such as shape, size, and morphology, depended on several factors, i.e., pH, temperature, reaction time, and concentrations of the reducing agent and silver salt^13,14^. Various kinds of plant extracts, such as oregano essential oil^15^, gelatin^16^, and green tea^17^, have been applied as bioreducing agents for the preparation of AgNPs. Recently, the use of bioreducing agents derived from wastes, such as agricultural^18^, food^19^, and beverage^20^ wastes, is a new trend for the green synthesis of AgNPs. Srikhao et al. prepared AgNPs using sugarcane leaf extract^18^. The existing phenolic compounds acted as bioreducing agents. The obtained AgNPs were used as a sensor to detect ammonia and H_2_O_2_. Spent coffee ground (SCG) is a phenol-rich waste that has been produced approximately 6 million tons/year^21^. SCG waste is considered a pollution hazard when it is discarded in landfills^22^. Consequently, the use of SCG and its derivatives has certain benefits, including value added to SCG waste and the reduction of costs in waste management. Based on the literature, most green-synthesized AgNPs with SCG have been applied as antibacterial agents^21,22^ and catalysts^23^. Therefore, the search for new alternative uses of AgNPs would increase their usefulness.

At present, paper-based sensors are widely used because of their simplicity, low cost, and high optical contrast for the colorimetric detection of chemical substances^24^. Furthermore, the use of paper-based sensors can be combined with a smartphone as a detector to improve analysis capability and portability. Yoo et al. successfully used a smartphone camera as a readout device to analyze paper-based sensors for glucose detection in the range of 0–10 mM. After glucose detection, the color of the paper-based sensor changed. This color change was recorded using a smartphone camera. Then, the red, green, and blue (RGB) program in the smartphone was applied to analyze the glucose concentration^25^.

Currently, there is a dearth of information on the preparation and characterization of paper-based sensors containing green-synthesized AgNPs. Hence, this study aimed to prepare AgNPs using an extract of SCG (ex-SCG) for coating paper. The coated paper was applied as a new paper-based sensor for H_2_O_2_ detection. The selectivity and long-term stability of P-AgNPs were investigated. The use of P-AgNPs combined with a smartphone was applied to analyze the H_2_O_2_ concentration.

## Materials and methods

### Material

SCG used in this work was collected from a local coffee shop in Khon Kaen, Thailand. Polyvinyl alcohol (PVA) with an average molecular weight in the range of 1700–1800 was purchased from Loba Chemie Pvt. Ltd., Mumbai, India. Silver nitrate (AgNO_3_, with a purity of 99.8%) was purchased from RCI Labscan Limited, Bangkok, Thailand. Whatman filter paper no. 1 (GE Healthcare UK Limited) was used as a substrate for the preparation of the paper-based sensor. Gallic acid (with a purity of 98%) and Folin–Ciocalteu’s reagent (with a density of 1.27 g/cm^3^, equivalent acid of 2 N) were purchased from Sigma Aldrich, Singapore, and Loba Chemie Pvt. Ltd., Mumbai, India, respectively. H_2_O_2_ commercial test strips (Peroxide 1000) were obtained from Quantofix, Germany. H_2_O_2_ with a concentration of 30% was purchased from Merck Schuchardt OHG, Kenilworth, NJ, USA.

### Preparation and characterization of ex-SCG

After obtaining SCG from the local coffee shop, it was first dried at a temperature of 80 °C for 24 h and then kept in a sealed plastic bag. ex-SCG was prepared using the SCG-to-deionized water ratio of 1:200 g/mL at a temperature of 95 °C for 5 min, according to the previous method of Trongchuen et al.^26^. The total phenolic content (TPC) of ex-SCG was determined following the Folin–Ciocalteu method^27^. The sample obtained using this method was analyzed by the Agilent Cary 60 UV–Vis spectrophotometer. The TPC of ex-SCG was reported as the gallic acid equivalent (GAE), which was determined to be 1.94 ± 0.16 mg GAE/g SCG.

### Preparation of paper-based sensor for H_2_O_2_ detection

PVA (2.5 g) was dissolved in 100 mL ex-SCG at a temperature of 90 °C for 60 min. Then, 1.2 mL AgNO_3_ solution at concentrations of 0, 50, 100, and 150 mM was added to the PVA solution and stirred at a temperature of 90 °C for another 15 h under reflux. The obtained mixture was cast on a filter paper using a doctor blade with a gap of 200 µm. Finally, the coated paper was dried at a temperature of 50 °C for 10 min.

### Characterization of the paper-based sensor for H_2_O_2_ detection

The UV–Vis spectra of green-synthesized AgNPs were analyzed by the Agilent Cary 60 UV–Vis spectrophotometer. X-ray diffraction (XRD) of paper coated with AgNPs was characterized by the SmartLab X-ray diffractometer (Rigaku, Japan) over diffraction angles of 10° to 80°. The diffractometer was equipped with a Cu Ka radiation source (wavelength *l* = 1.542 Å) operated at the scan rate of 0.01° at 40 kV and 30 mA. Transmission electron microscopy (TEM) images of the green-synthesized AgNPs at different AgNO_3_ concentrations were obtained using the TECNAI G2 20 transmission electron microscope (FEI, Hillsboro, OR, USA). The surface of P-AgNPs was analyzed and high-resolution energy-dispersive X-ray spectroscopy (EDX) mapping was conducted using a scanning electron microscope (Hitachi Miniscope Model TM-3000). All samples were coated with gold using an ion sputtering device. The sample solution was deposited on 400-mesh carbon-coated Cu grids and dried at room temperature for 24 h. The TEM images were taken at 200 kV accelerating voltage. The attenuated total reflectance Fourier transform infrared (ATR-FTIR) spectra (TENSOR 27, Bruker, Billerica, MA, USA) were used to examine the functional groups of the paper coated with AgNPs. All spectra were obtained at 4000–600 cm^−1^ with 64 scans at a resolution of 2 cm^−1^.

### Colorimetric assay of H_2_O_2_

Paper-based sensors (with a circular area diameter of 6 mm) were immersed in 50 mL H_2_O_2_ standard solution with concentrations of 0–6000 mg/L and incubated at room temperature for 45 s. Then, the paper-based sensors were placed under a light-emitting diode (60 W) lamp. The color images of the paper-based sensors were taken by a smartphone (iPhone XR). The obtained images were analyzed by quantification using the ImageJ Version 1.8 software. The average color intensities of RGB were obtained to calculate the RGB distance (∆RGB) value according to Eq. (1)^28,29^:

1 $$\vartriangle {\text{RGB }}= \sqrt {{\text{(R} - {\text R}}_{{0}} {)}^{{2}} +{\text{ (G}} - {\text {G}}_{{0}} {)}^{{2}} +{\text{ (B}} - {\text {B}}_{{0}} {)}^{{2}} } ,$$

where the R, G, and B values are values after immersing the paper sensors in known H_2_O_2_ solutions and the subscript 0 refers to the values before immersion. Then, the values of ∆RGB and the known H_2_O_2_ concentrations were curve-fitted to obtain the relationship needed to calculate the H_2_O_2_ concentration for a known ∆RGB.

### Long-term stability test of the sensor

The sensor samples were kept in a plastic box and then stored at 25 °C under dark conditions for 100 days. The long-term stability was tested by examining the efficiencies of the sensors according to a colorimetric assay of H_2_O_2_.

## Results and discussions

### Characterization of green-synthesized AgNPs

**Figure 1:**
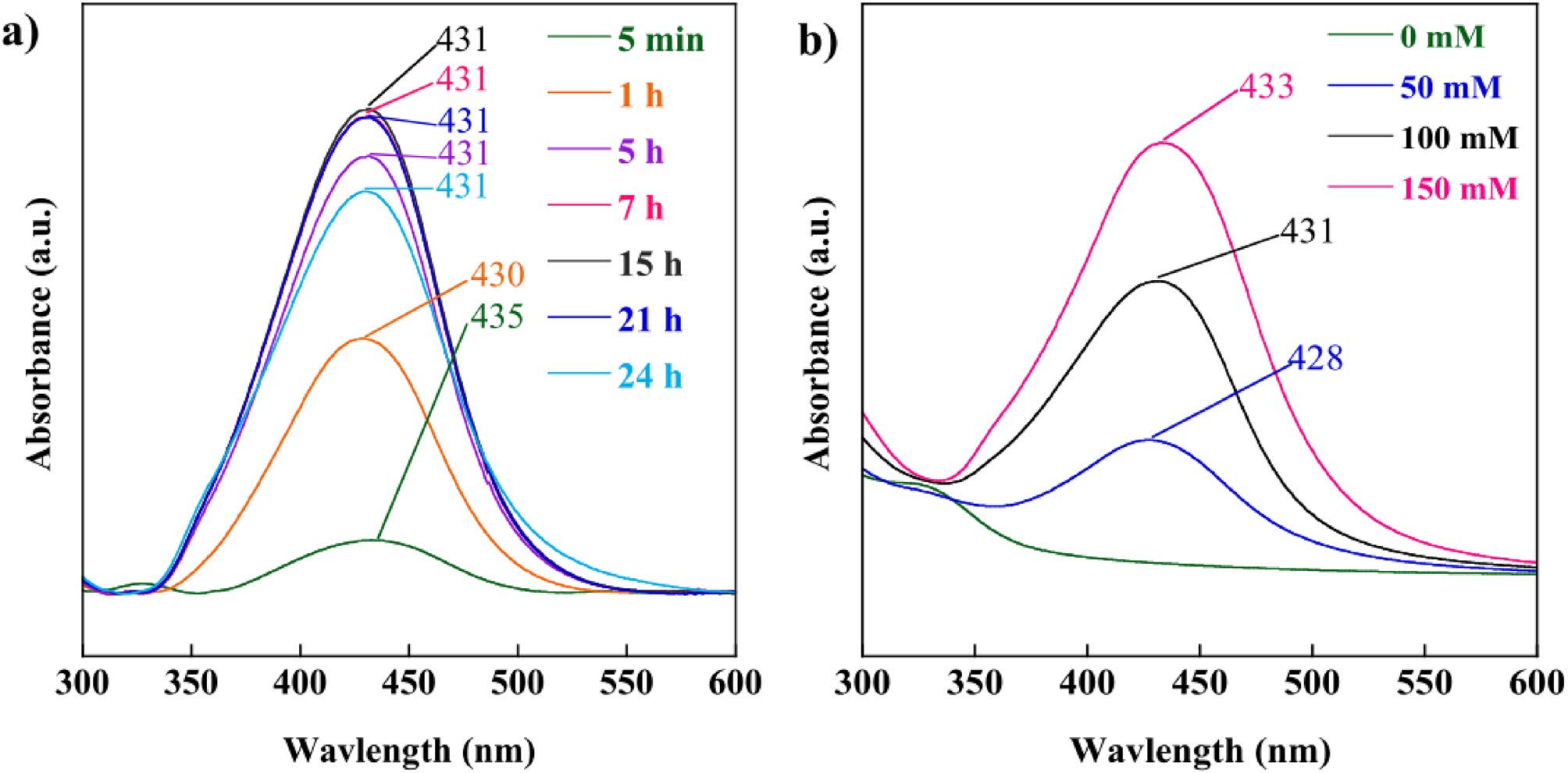
UV–Vis spectra of AgNPs at (**a**) different reaction times and (**b**) different concentrations.

AgNPs are well known to show their unique optical properties for SPR in the range of 400–500 nm^30^. The SPR bands of green-synthesized AgNPs with ex-SCG at various synthesis times are depicted in Fig. 1a,b. After synthesis for 5–60 min, the SPR band at 431–435 nm was observed, as shown in Fig. 1a. This phenomenon indicated AgNP formation. The intensity of the SPR band increased with the increase in synthesis time from 5 min to 15 h. The increase in peak intensity was attributed to the continued reduction of silver ions to form AgNPs^28,31^. After the synthesis of AgNPs for 15 h, the intensity of the SPR band decreased possibly because of the agglomeration of AgNPs at longer synthesis times^18^. Furthermore, a blue shift in the wavelength of 435 nm for the synthesis time of 5 min to 431 nm for the synthesis time of 15 h was observed. The blue shift of SPR for NPs indicated a decrease in particle size^20^. The synthesis time of 15 h was determined to be the optimum condition. Therefore, the AgNPs green-synthesized for 15 h were used as paper coating. Figure 1b shows the UV–Vis spectra of AgNPs at AgNO_3_ concentrations of 0, 50, 100, and 150 mM. The peak of green-synthesized AgNPs with ex-SCG was detected in the range of 428–433 nm, whereas the absorption spectra of ex-SCG (0 mM AgNO_3_) did not show any characteristic peaks in this range. The peak intensity of AgNPs increased with the increase in AgNO_3_ concentration from 50 to 150 mM. Taesuwan et al.^32^ proposed that the increase in peak intensity can be attributed to the high yield of AgNP formation because of more available reactants. The red shift of SPR from 428 nm for synthesized AgNPs with 50 mM to 433 nm for synthesized AgNPs with 150 mM was observed with the increase in AgNO_3_ concentration. Amirjani et al.^33^ reported that the red shift of SPR indicated the increase in the size of AgNPs.

**Figure 2:**
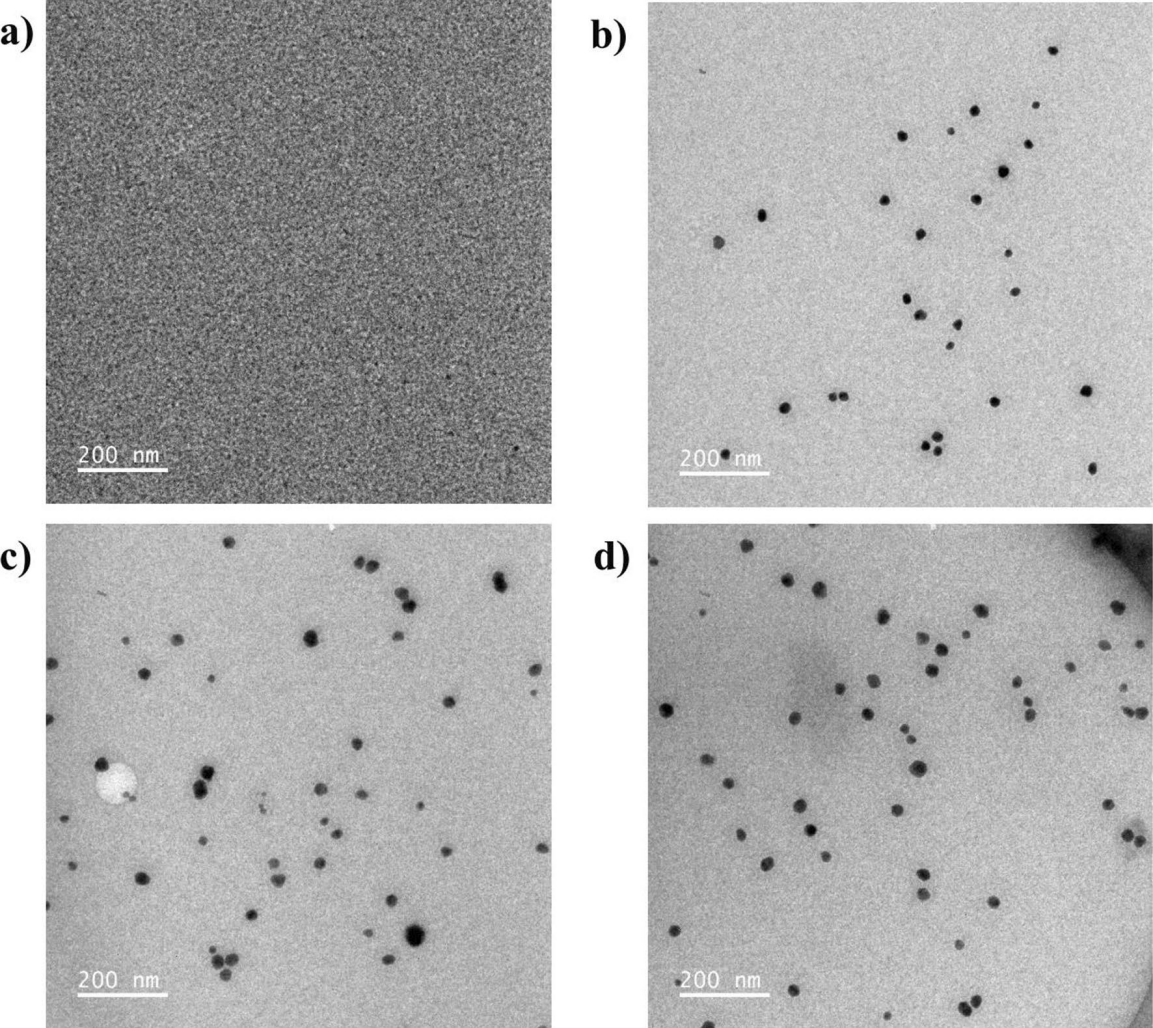
TEM micrographs of AgNPs at different AgNO_3_ concentrations: (**a**) 0 mM, (**b**) 50 mM, (**c**) 100 mM, and (**d**) 150 mM.

**Figure 3:**
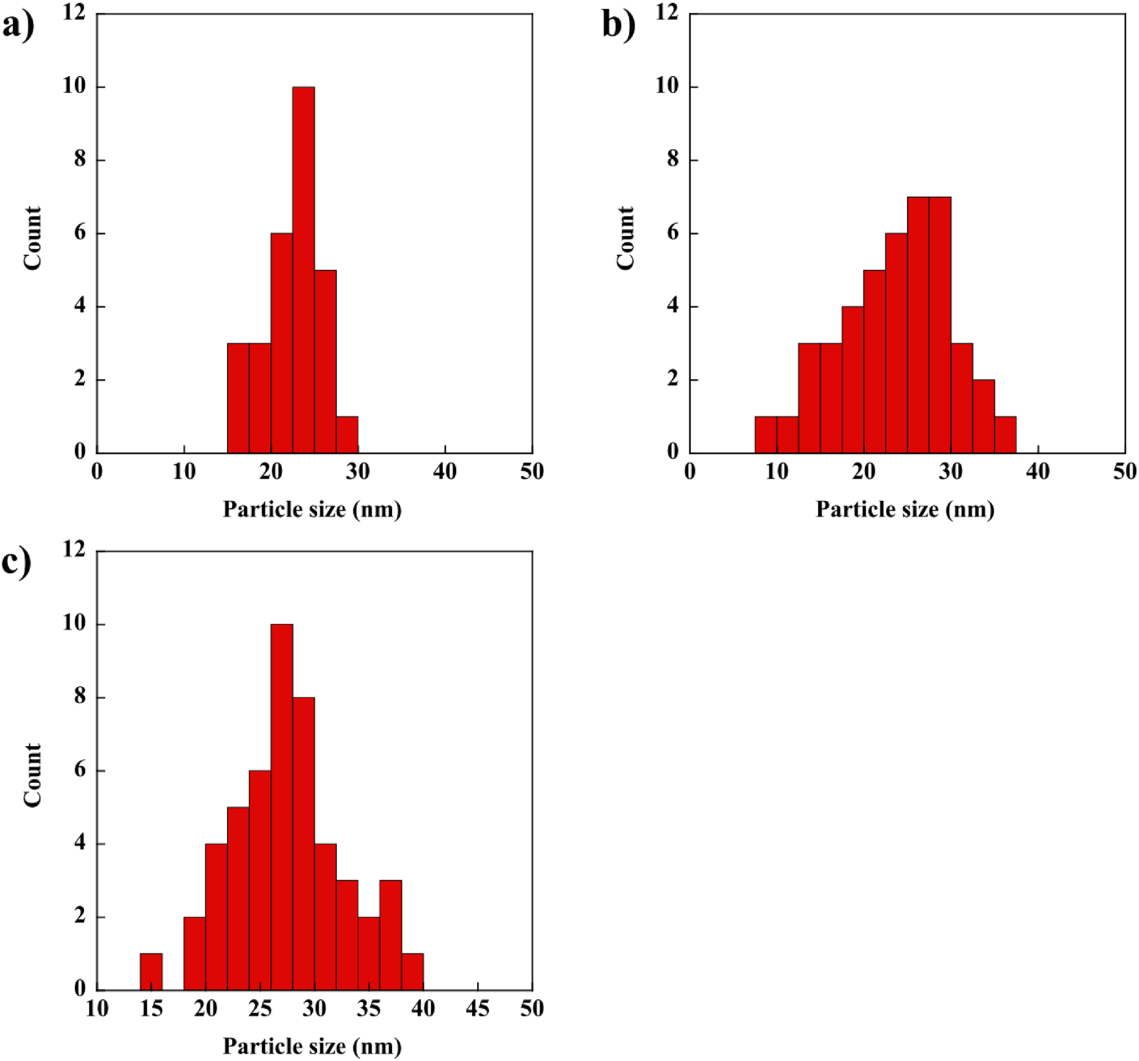
Size distribution of AgNPs at different AgNO_3_ concentrations: (**a**) 50 mM, (**b**) 100 mM, and (**c**) 150 mM.

The morphology and size of AgNPs were analyzed using TEM, as depicted in Figs. 2a–d and 3a–c, respectively. A spherical shape was observed for all AgNPs at different AgNO_3_ concentrations. Furthermore, the amount of AgNPs increased with the increase in AgNO_3_ concentration from 50 to 150 mM. As illustrated in Fig. 3a–c, the particle size of AgNPs ranged from 8.64 to 38.99 nm. For 50 mM AgNO_3_, the size of AgNPs ranged from 15.37 to 27.98 nm. For 100 mM AgNO_3_, the size of AgNPs ranged from 8.64 to 35.68 nm. For 150 mM AgNO_3_, the size of AgNPs ranged from 15.40 to 38.99 nm. These results could be explained by the fact that, at high AgNO_3_ concentrations, the rate of spontaneous nucleation increased the growth rate of AgNPs. Then, large numbers of nuclei were formed during burst nucleation. The formation of a large amount of AgNPs with free and high surface tension leads to larger particles^34,35^. The size distributions and shapes of AgNPs in ex-SCG solution should be investigated using the dynamic light scattering technique to consolidate the material characterization in further study.

**Figure 4:**
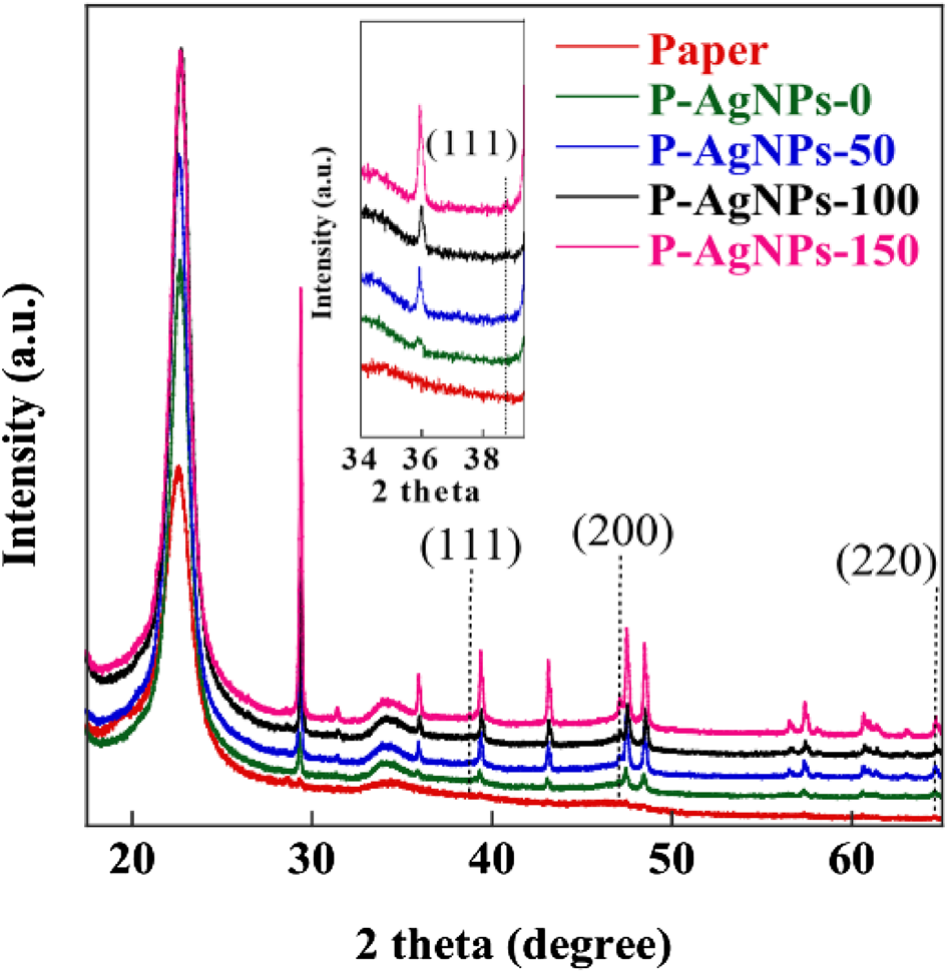
XRD patterns of the filter paper and P-AgNPs.

The crystalline nature of P-AgNPs was investigated using XRD analysis. Figure 4 shows the XRD patterns of pristine paper at 22.52°, which corresponds to the (220) crystallographic planes of the monolithic cellulose type I^36,37^. The paper coated with PVA/ex-SCG exhibited the characteristic peaks at 2*θ* = 29.50° to 67°. These peaks were attributed to the structures of PVA and the organic components of ex-SCG. Malik et al.^30^ detected a semicrystalline and a small amorphous peak of PVA at 19.5° and 39° to 40°, respectively. For ex-SCG, the peaks in the range of 20° to 80° can be assigned to chlorogenic acid^38^, caffeic acid^39^, and the bioorganic phase of ex-SCG^40^. In the case of AgNPs, the XRD pattern of Ag crystals consisted of four peaks in the range 2*θ* = 20° to 80°, which correspond to reflections of the (111), (200), (220), and (311) planes from the face-centered cubic unit cell. P-AgNPs-50, P-AgNPs-100, and P-AgNPs-150 showed weak reflections of the (111) plane at 38.80° and (200) plane at 47.05°. Furthermore, the peak intensity at 64.72° (220) increased with the increase in AgNP concentration. The XRD profile data of P-AgNPs corresponded to the standard JCPDS file no. 04-0783 and was consistent with the crystal planes of AgNPs reported by Satyanarayana^41^. The overlap of the AgNP characteristic peaks and those of other materials resulted in weak XRD signals^42^. The average crystallite size values of AgNPs can be calculated from the XRD pattern using the Scherrer equation, as expressed in Eq. (2). The average crystallite size was determined to be 19.09 nm for P-AgNPs-50, 24.58 nm for P-AgNPs-100, and 32.66 nm for P-AgNPs-150.

2 $${\text{D}} = \frac{{{\text{k}}\lambda }}{{\beta {{{\rm cos}\theta }}}}$$

where *D* is the crystallite size (nm), *k* is the Debye–Scherrer constant (0.89), *λ* is the X-ray wavelength, *β* is the line broadening in radians obtained from the full width at half maximum, and *θ* is the Bragg angle or peak position (radians).

**Figure 5:**
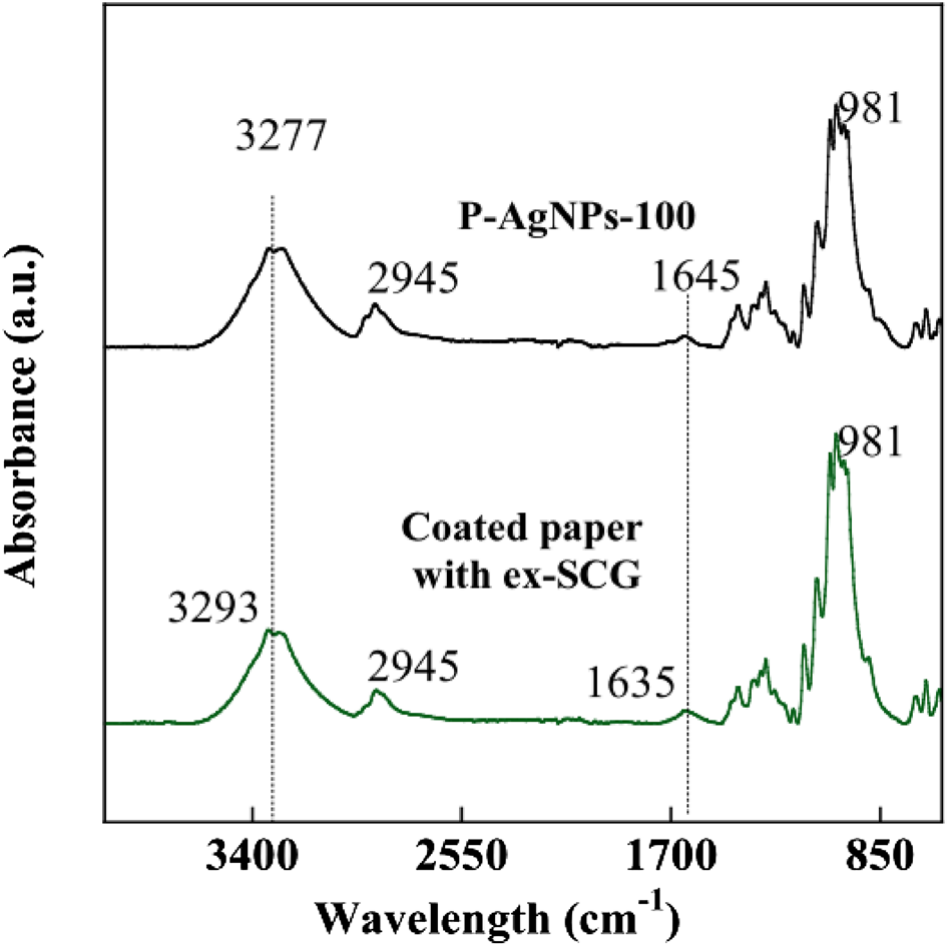
ATR-FTIR spectra of the paper coated with ex-SCG and P-AgNPs-100.

ATR-FTIR analysis was performed to detect the functional groups of bioreducing agents for the green synthesis of AgNPs as depicted in Fig. 5. The ATR-FTIR spectra of coated paper with ex-SCG were detected at 981 cm^−1^ (C=C bending), 1635 cm^−1^ (C=C stretching), 2945 cm^−1^ (C–H stretching), and 3293 cm^−1^ (O–H stretching). For P-AgNPs, the shift of peaks was only observed when compared with the spectra of coated paper with ex-SCG. The peaks at 1635 and 3293 cm^−1^ shifted to 1645 and 3277 cm^−1^, respectively. These shifted peaks indicated that the bioreducing agent could reduce silver ions to AgNPs and form a layer on the AgNP surface^43^. By integrating the hypotheses of previous reports^44–46^ with the results of the present study, a possible mechanism for the green synthesis of AgNPs using phenolic compounds in ex-SCG can be proposed, as illustrated in Supplementary Fig. S1. The main phenolic compounds, such as chlorogenic acid, ferulic acid, caffeic acid, and p-coumaric acid, could be obtained by extraction of SCG with water^47,48^. In green synthesis, phenolic compounds could release electrons to reduce silver ions and Ag cation reduction could be coupled with the oxidation of the hydroxyl group of phenolic compounds^44,49^.

### Optimization of the detection conditions

**Figure 6:**
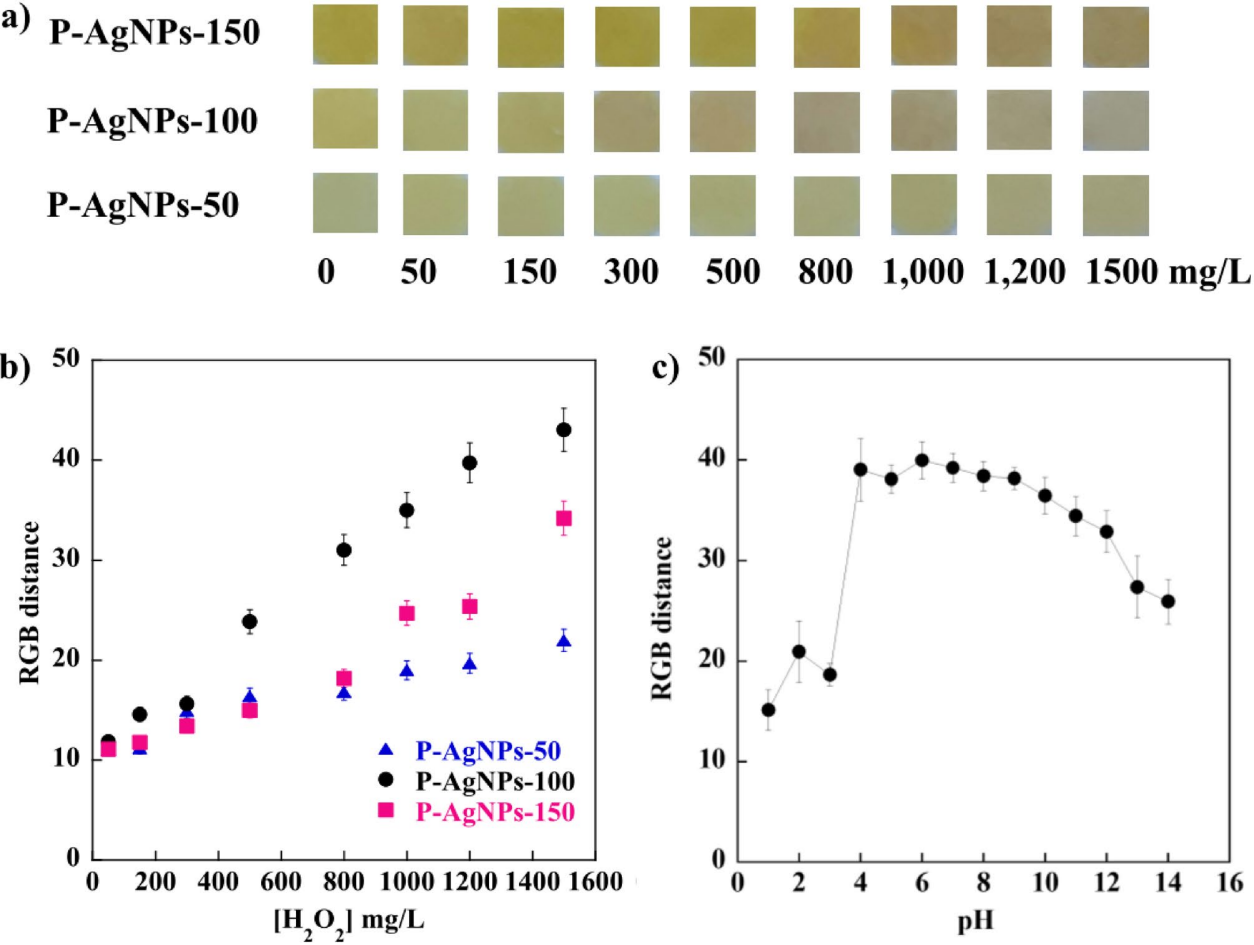
(**a**) Digital images of the test P-AgNPs in the presence of different H_2_O_2_ concentrations, (**b**) analytical curves for H_2_O_2_ detection by P-AgNPs, and (**c**) analytical curves for H_2_O_2_ detection by P-AgNPs-100 at different pH values.

The necessary detection conditions, such as the AgNP concentration and pH value of the H_2_O_2_ solution, were optimized using the ∆RGB values as a criterion. Figure 6a,b show that P-AgNPs-100 has the highest response to the color change and ∆RGB values, respectively, possibly because of the large amount of synthesized AgNPs with 100 mM AgNO_3_. Although the obtained amount of AgNPs was lower than the synthesized AgNPs with 150 mM AgNO_3_, the obtained AgNPs from 100 mM AgNO_3_ still had a more uniform and narrower size distribution, as observed in the UV–Vis spectral analysis in Fig. 1b and TEM analysis in Fig. 3. Furthermore, the EDX images in Supplementary Fig. S2 illustrated that P-AgNPs-100 had a homogeneous distribution of AgNPs on the surface, whereas P-AgNPs-150 had agglomerated AgNPs. The Ag content on the surface increased slightly with the increase in AgNO_3_ content. Figure 6c shows the effect of pH on H_2_O_2_ detection using P-AgNPs-100. The RGB values for pH values of 1–3, 4–9, and 10–14 were in the ranges of 15.15–20.93, 38.15–39.03, and 25.93–36.46, respectively. The maximum intensity of RGB values was observed in the pH range of 4–9. Farrokhnia et al.^50^ reported that H_2_O_2_ preferred to decompose in alkaline media. The intensity of RGB decreased at pH values of 1–3. For acidic media, the silver ions produced from AgNPs in the presence of H_2_O_2_ might not further oxidize and the reaction between hydronium ion and H_2_O_2_ at acidic pH enabled H_2_O_2_ detection by AgNPs^51^. A higher ∆RGB value indicated a higher detection response of the paper-based sensor^24,52^. Therefore, the optimum pH range for H_2_O_2_ detection was 4–9. A similar pH range of 5–7 for H_2_O_2_ detection using AgNPs was reported by Prapaporn et al.^53^.

### H_2_O_2_ responsiveness of P-AgNPs-100

**Figure 7:**
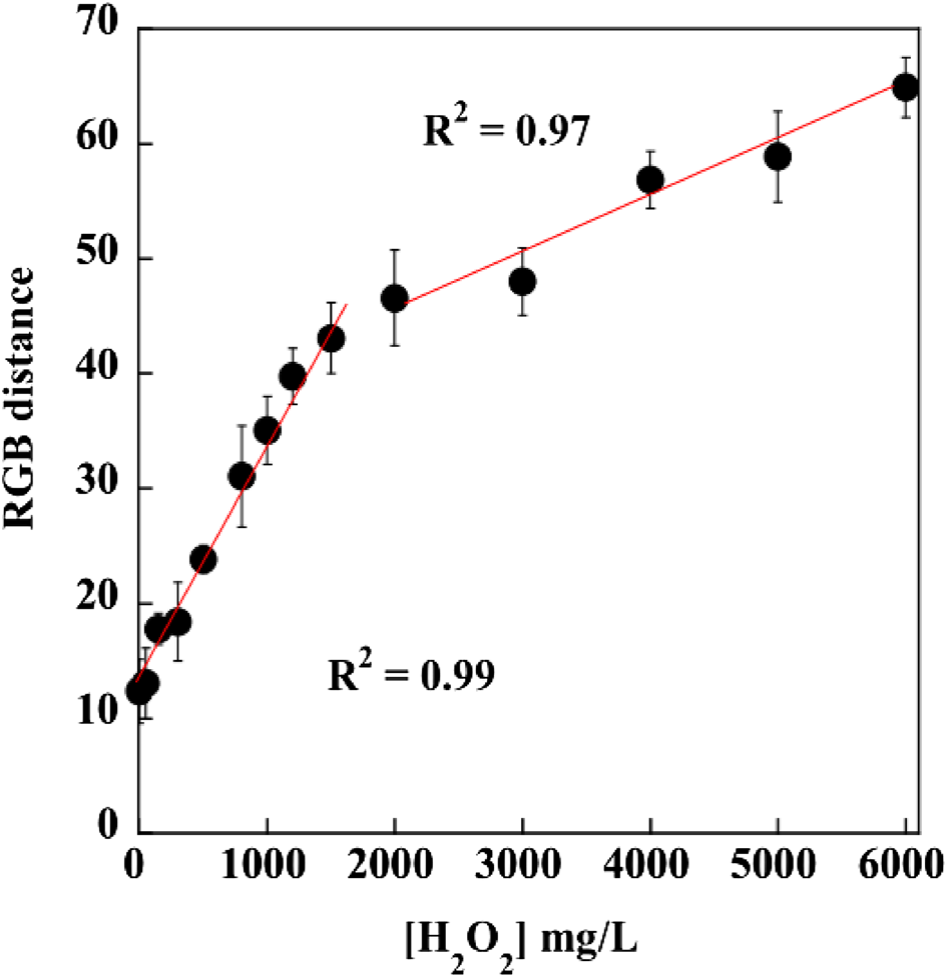
Calibration curve plotting the signal intensity of color detected by P-AgNPs-100 integrated with the ImageJ software versus the H_2_O_2_ concentration.

The colorimetric sensing capability of P-AgNPs-100 for H_2_O_2_ was determined, as illustrated in Fig. 7. After the H_2_O_2_ solution was deposited on P-AgNPs-100, color change with different shades depending on the H_2_O_2_ concentration was detected, which was attributed to the destruction of P-AgNPs-100 by the oxidation capability of H_2_O_2_. The AgNPs were oxidized to silver ions^54^. The possible reaction mechanism of AgNPs and H_2_O_2_ is expressed as Eq. (3):

3 $${\text{2Ag}} + {\text{H}}_{{2}} {\text{O}}_{{2}} \to {\text{ 2Ag}}^{ + } + {\text{ 2OH}}^{ - } $$

The ∆RGB values increased linearly with the increase in H_2_O_2_ concentrations in the range of 50–1500 and 1500–6000 mg/L. Hence, the plot of ∆RGB values versus H_2_O_2_ concentrations was considered in the two ranges. The calibration curve plotting the signal intensity of color detected by P-AgNPs-100 integrated with the ImageJ software versus the H_2_O_2_ concentration.

The regression equation was *Y* = 13.249 + 0.021*X*, *R*^2^ = 0.99, for the H_2_O_2_ concentration range of approximately 50–1500 mg/L and *Y* = 35.942 + 0.0048*X*, *R*^2^ = 0.97, for the H_2_O_2_ concentration range of approximately 1500–6000 mg/L, where *x* is the H_2_O_2_ concentration (mg/L). The limit of detection (LOD) of response was calculated using Eq. (4)^50^:

4 $${\text{LOD }} = {\text{ average response of the blank }} + \, \left( {{3 } \times {\text{ standard deviation of the blank}}} \right)$$

The LOD of P-AgNPs-100 was 1.26 mmol/L. The obtained LOD value for H_2_O_2_ detection was relatively higher than those for paper-based sensors, which ranged from 1.75 to 100 µmol/L^11,55,56^. Notably, P-AgNPs-100 can be used as a sensor to detect H_2_O_2_ in the preparation of sanitizer agents, which normally contain H_2_O_2_ in the range of 97.06–11,426.47 mmol/L for household sanitizers^57^ and 8.16–970 mmol/L for fruit sanitizers^2^.

### Effect of interferents on the selective determination of H_2_O_2_ using the P-AgNPs-100 sensor

**Figure 8:**
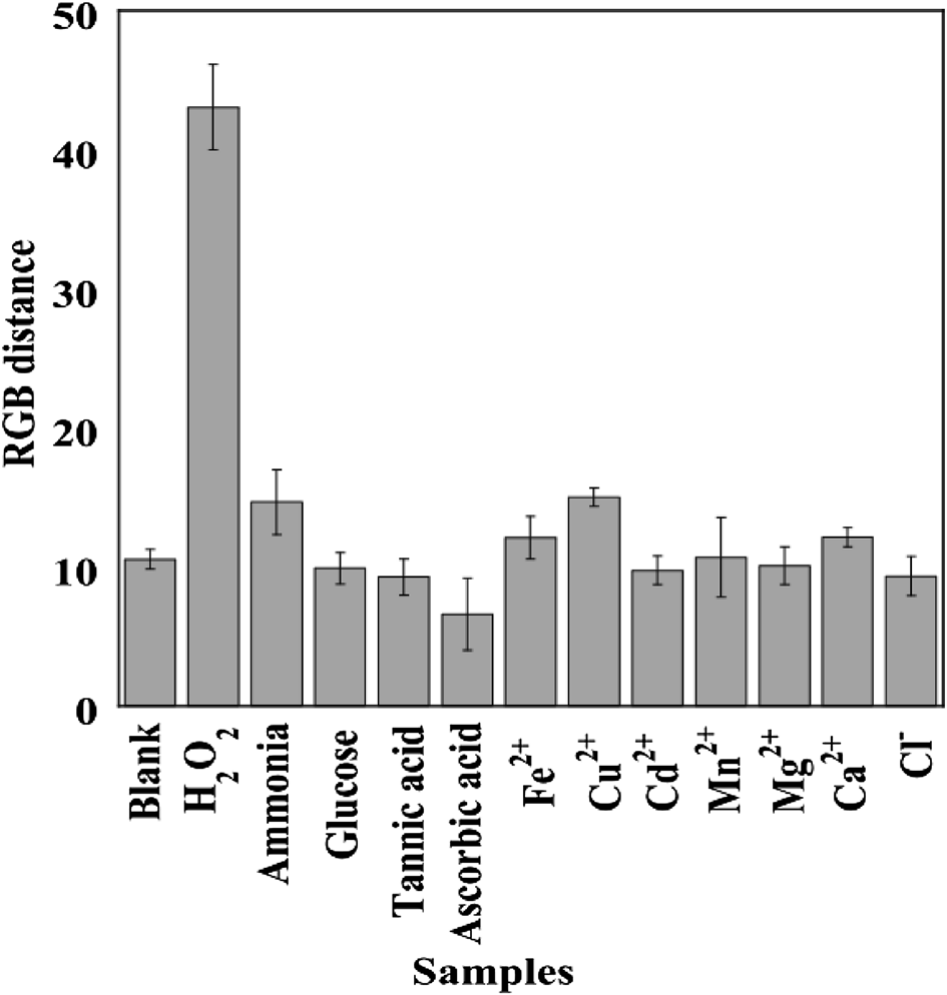
Signal intensity of the color detected by P-AgNPs-100 with different metal ions and anions.

The selectivity test of the paper-based sensor was conducted by depositing metal ions and anions in the sensor sample. The color change of the sensor referred to the tolerance limit^24^. Various types of metal ions and anions, viz., Fe^2+^, Cu^2+^, Cd^2+^, Mn^2+^, Mg^2+^, Ca^2+^, Cl^−^, ammonia, glucose, tannic acid, and ascorbic acid, were used to determine the selectivity of P-AgNPs-100, as shown in Fig. 8. Notably, the ∆RGB values obtained with H_2_O_2_ were higher than those with other agents, indicating that the proposed P-AgNPs100 exhibited high selectivity for H_2_O_2_ detection.

### Application of P-AgNPs-100 in sanitizer preparation for H_2_O_2_ detection

To validate the applicability of P-AgNPs-100 for H_2_O_2_ detection, the H_2_O_2_ detection capability of P-AgNPs-100 was tested and compared with that of a commercial strip sensor. The recovery value from the actual value of H_2_O_2_ was calculated according to Eq. (5)^58^:

5 $${\text{Recovery }}\left( \% \right) \, = \, \left( {{\text{found value}}/{\text{added value}}} \right) \, \times { 1}00$$

The recovery values of H_2_O_2_ at different concentrations in tap water are summarized in Table 1. The recovery values of P-AgNPs-100 and a commercial strip sensor ranged from 97.55 to 101.21% and from 105.37 to 106.49%, respectively. Notably, P-AgNPs-100 had recovery values comparable with those of a commercial strip sensor.

**Table 1:** Recoveries (%) achieved for [H_2_O_2_]-spiked sample in tap water via the proposed sensor compared with the commercial strip sensor.

Matrix	[H_2_O_2_] added (mg/L)	Proposed sensor			Commercial H_2_O_2_ strip sensor		
		[H_2_O_2_] found (mg/L)	RSD (%) (*n* = 5)	Recovery (%)	[H_2_O_2_] found (mg/L)	RSD (%) (*n* = 5)	Recovery (%)
Tap water	150	143.33	7.76	97.55	158.26	1.15	105.51
Tap water	300	300.61	7.97	100.20	319.48	7.07	106.49
Tap water	500	506.05	9.65	101.21	526.86	4.34	105.37

Furthermore, the H_2_O_2_ concentration in various types of sanitizers was tested using P-AgNPs-100. Three sanitizers, viz., fruit sanitizer, water for household settings^57^, and H_2_O_2_-based spray (against coronavirus) for dental settings^59^, were prepared with different H_2_O_2_ concentrations. These samples were spiked with known amounts of H_2_O_2_, and the recovery values were determined, as shown in Table 2. The recoveries of H_2_O_2_ at different concentrations were determined to be more than 99% with good precision (RSD ≤ 8%). The recovery values and RSD of P-AgNPs-100 were in an acceptable range, as reported in previous studies^60,61^. The obtained results indicated that P-AgNPs-100 exhibited high efficiency for the determination of H_2_O_2_ in real samples.

**Table 2:** Recoveries (%) achieved for [H_2_O_2_] in various sanitizers via P-AgNPs-100.

Type of sanitizer	Added [H_2_O_2_] (%)	[H_2_O_2_] found (%)	Recovery (%)	RSD (%) (*n* = 5)
Orange juice + 0.025% H_2_O_2_	0.025	0.0249	99.82	7.31
	0.025^a^	0.0251^a^	100.27^a^	6.80^a^
Water for household settings (0.3% H_2_O_2_)	0.3	0.298	99.28	4.45
H_2_O_2_-based spray (against coronavirus) for dental settings (0.5% H_2_O_2_)	0.5	0.499	99.90	3.53

^a^Commercial test strips.

### Long-term stability of P-AgNPs-100

**Figure 9:**
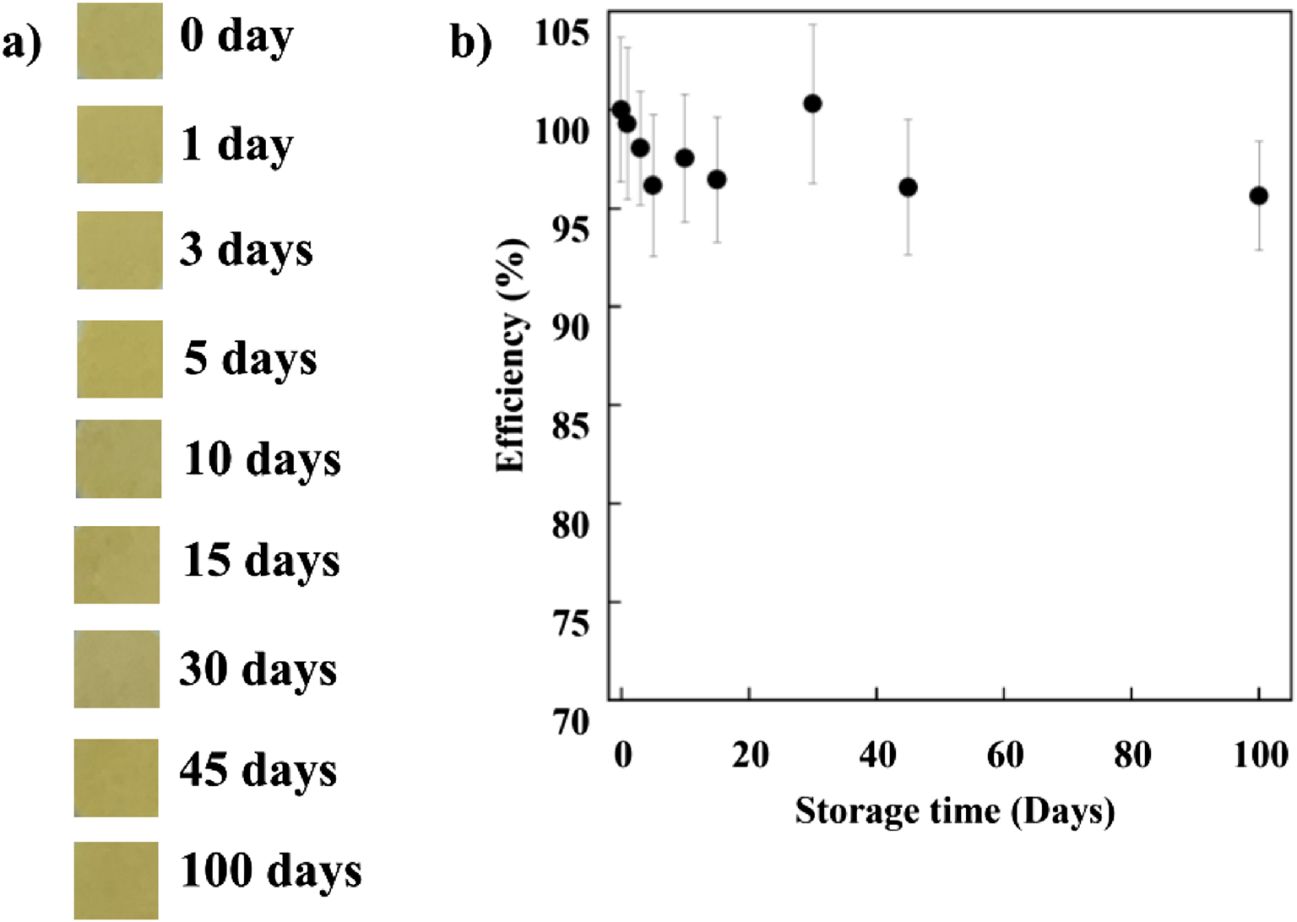
(**a**) Digital images of P-AgNPs-100 during storage and (**b**) storage stability of P-AgNP-100 for H_2_O_2_ detection.

The long-term stability of the sensor during storage was a necessary factor in practical application^62^. After P-AgNPs-100 was stored at room temperature under dark conditions for 100 days, no apparent change was detected in the sample, as illustrated in Fig. 9a. Furthermore, more than 95% of the initial activity was retained within 100 days, as depicted in Fig. 9b, possibly because of the oxidation of AgNPs by atmospheric oxygen^63^. However, the efficiency of P-AgNPs-100 after storage was comparable with that of other sensors in previous reports, as summarized in Table 3.

**Table 3:** Long-term stability in efficiency (%) and storage conditions of the previous reports for H_2_O_2_ paper-based sensors and other solid-phase sensors.

No	H_2_O_2_ sensor	Lifetime and storage condition	Efficiency (%)	References
1	HRP-enzyme/poly(ANI-co-AA) composite platforms	30 days: thermostatic oven at 37 °C	95%	^64^
2	Nanoceria-embedded paper strip for H_2_O_2_	20 days storage conditions at room temperature (RT), 4 °C, and − 20 °C	> 90% at RT	^65^
3	Electrochemical sensor based on ZIF-67/CNFs/GCE	30 days storage in 0.1 M NaOH solution	92.6%	^66^
4	Pt NPs/RGO-CS-Fc biosensor	22 days in a humid environment at 4 °C	80%	^67^
5	Mediated turnip tissue paper-based amperometric biosensor	25 days at 4 °C	~ 70%	^68^
6	P-AgNPs-100	100 days: room temperature (~ 30 °C), darkness	> 95%	This work

## Conclusions

We have developed an H_2_O_2_ paper-based sensor using agricultural waste (i.e., ex-SCG) for the green synthesis of AgNPs. The optimum conditions for the preparation of P-AgNPs were determined to be 100 mM AgNO_3_ and 15 h reaction time. The combination of the paper-based sensor with smartphone readout is simple, efficient, and inexpensive. The detection limit of H_2_O_2_ for P-AgNPs-100 was observed at 1.26 mM. P-AgNPs-100 also had recovery values of H_2_O_2_ comparable with those of a commercial strip sensor. P-AgNPs-100 showed acceptable long-term stability of H_2_O_2_ detection with an efficiency of 95.62% when stored for 100 days. Furthermore, P-AgNPs-100 exhibited excellent recovery of H_2_O_2_ in the sanitizers and tap water, which confirmed possible application for H_2_O_2_ detection in real samples.

## Supplementary Information

Supplementary Figures.

## Data Availability

The datasets used and/or analyzed during the current study are available from the corresponding author on reasonable request.
