# Supplementary material for: Green synthesis of silver nanoparticles using the extract of spent coffee used for paper-based hydrogen peroxide sensing device
Source: Sci Rep. 2022 Nov 22;12:20099. doi: 10.1038/s41598-022-22067-6 (PMC9684408; doi:10.1038/s41598-022-22067-6)
Supplement: Supplementary file 1 — Supplementary Figures. [file 41598_2022_22067_MOESM1_ESM.docx]

**Supplementary Information**

**Green synthesis of silver nanoparticles using the extract of spent coffee used for paper-based hydrogen peroxide sensing device**


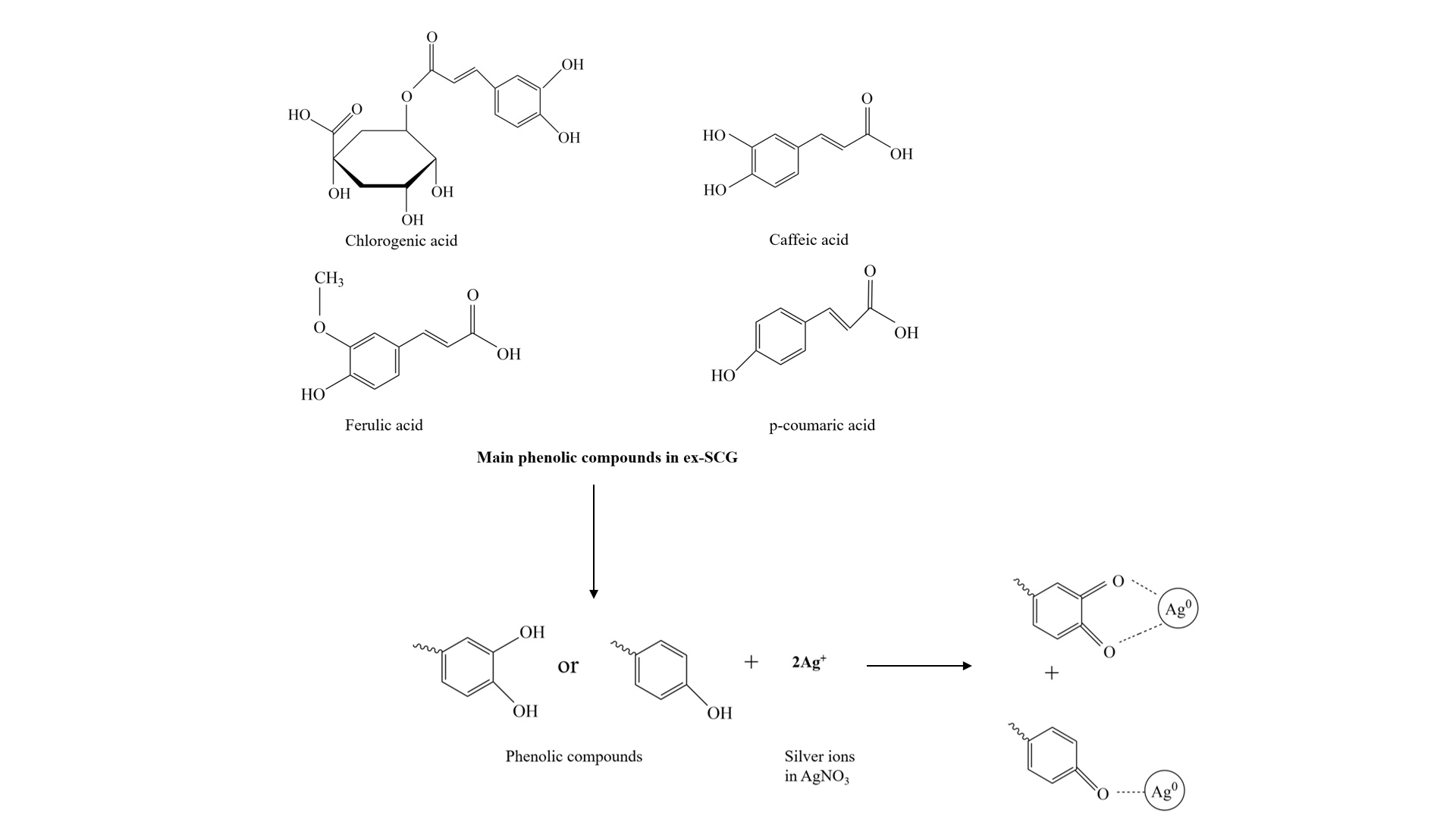


**Fig. S1** Possible mechanism for the green synthesis of AgNPs using phenolic compounds in ex-SCG**.**


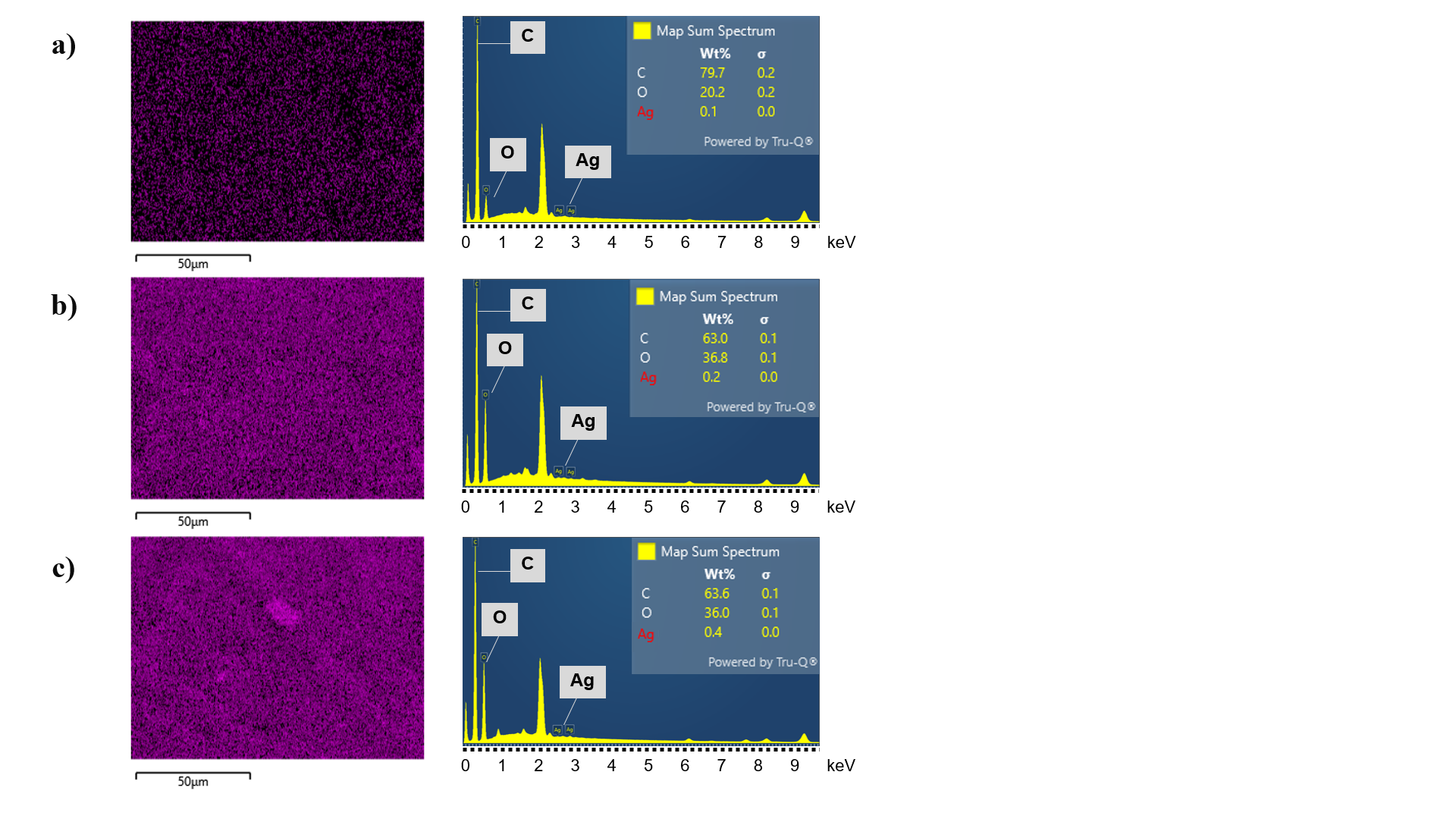


**Fig. S2** EDX spectra of P-AgNPs samples.
